# A novel machine learning-based cancer-specific cardiovascular disease risk score among patients with breast, colorectal, or lung cancer

**DOI:** 10.1093/jncics/pkaf016

**Published:** 2025-01-30

**Authors:** Nickolas Stabellini, Omar M Makram, Harikrishnan Hyma Kunhiraman, Hisham Daoud, John Shanahan, Alberto J Montero, Roger S Blumenthal, Charu Aggarwal, Umang Swami, Salim S Virani, Vanita Noronha, Neeraj Agarwal, Susan Dent, Avirup Guha

**Affiliations:** Division of Cardiology, Department of Medicine, Medical College of Georgia at Augusta University, Augusta, GA 30912, United States; Case Western Reserve University School of Medicine, Case Western Reserve University, Cleveland, OH 44106, United States; Department of Hematology-Oncology, University Hospitals Seidman Cancer Center, Cleveland, OH 44106, United States; Faculdade Israelita de Ciências da Saúde Albert Einstein, Hospital Israelita Albert Einstein, São Paulo, SP 05652-900, Brazil; Division of Cardiology, Department of Medicine, Medical College of Georgia at Augusta University, Augusta, GA 30912, United States; Cardio-Oncology Program, Medical College of Georgia at Augusta University, Augusta, GA 30912, United States; Division of Cardiology, Department of Medicine, Medical College of Georgia at Augusta University, Augusta, GA 30912, United States; Cardio-Oncology Program, Medical College of Georgia at Augusta University, Augusta, GA 30912, United States; School of Computer and Cyber Sciences, Augusta University, Augusta, GA 30912, United States; Cancer Informatics, Seidman Cancer Center at University Hospitals of Cleveland, Cleveland, OH 44106, United States; Case Western Reserve University School of Medicine, Case Western Reserve University, Cleveland, OH 44106, United States; Johns Hopkins Ciccarone Center for the Prevention of Cardiovascular Disease, Baltimore, MD 21287, United States; Head & Neck and Thoracic Cancers section, Department of Hematology-Oncology, University of Pennsylvania, Philadelphia, PA 19104, United States; Division of Oncology, Department of Internal Medicine at Huntsman Cancer Institute, University of Utah, Salt Lake City, UT 84112, United States; Aga Khan University, Karachi 30270 - 00100, Pakistan; Department of Medical Oncology, Tata Memorial Center, Mumbai 400012, India; Division of Oncology, Department of Internal Medicine at Huntsman Cancer Institute, University of Utah, Salt Lake City, UT 84112, United States; Wilmot Cancer Institute, Department of Medicine, University of Rochester, Rochester, NY 14642, United States; Division of Cardiology, Department of Medicine, Medical College of Georgia at Augusta University, Augusta, GA 30912, United States; Cardio-Oncology Program, Medical College of Georgia at Augusta University, Augusta, GA 30912, United States

## Abstract

**Background:**

Cancer patients have up to a 3-fold higher risk for cardiovascular disease (CVD) than the general population. Traditional CVD risk scores may be less accurate for them. We aimed to develop cancer-specific CVD risk scores and compare them with conventional scores in predicting 10-year CVD risk for patients with breast cancer (BC), colorectal cancer (CRC), or lung cancer (LC).

**Methods:**

We analyzed adults diagnosed with BC, CRC, or LC between 2005 and 2012. An machine learning (ML) Extreme Gradient Boosting algorithm ranked 40-50 covariates for predicting CVD for each cancer type using SHapley Additive exPlanations values. The top 10 ML-predictors were used to create predictive equations using logistic regression and compared with American College of Cardiology (ACC)/American Heart Association (AHA) Pooled Cohort Equations (PCE), Predicting Risk of cardiovascular disease EVENTs (PREVENT), and Systematic COronary Risk Evaluation-2 (SCORE2) using the area under the curve (AUC).

**Results:**

We included 10 339 patients: 55.5% had BC, 15.6% had CRC, and 29.7% had LC. The actual 10-year CVD rates were: BC 21%, CRC 10%, and LC 28%. The predictors derived from the ML algorithm included cancer-specific and socioeconomic factors. The cancer-specific predictive scores achieved AUCs of 0.84, 0.76, and 0.83 for BC, CRC, and LC, respectively, and outperformed PCE, PREVENT, and SCORE2, increasing the absolute AUC values by up to 0.31 points (with AUC ranging from 0 to 1). Similar results were found when excluding patients with cardiac history or advanced cancer from the analysis.

**Conclusions:**

Cancer-specific CVD predictive scores outperform conventional scores and emphasize the importance of integrating cancer-related covariates for precise prediction.

## Introduction

Cardiovascular disease (CVD) is the leading cause of death globally in the general population and among cancer survivors.^1-5^ Patients with cancer have up to a 3-fold higher risk for atherosclerotic cardiovascular diseases (ASCVD) and 1.5-fold higher risk for heart failure (HF) than the general population.^1^^,^^2^ The numbers of cancer survivors have been consistently increasing over the years with increasing burden of other comorbid conditions that are not cancer related.^3-5^

The clinical decision making for allocating primary prevention interventions is usually dependent on the estimated 10-year cardiovascular risk scores of each individual. Several scores were developed to estimate the cardiovascular risk, for example, the American College of Cardiology (ACC)/American Heart Association (AHA) pooled cohort equations (PCE), the Predicting Risk of cardiovascular disease EVENTs (PREVENT) score, and the SCORE2 risk score.^6-9^ However, these scores were developed for the general population without explicitly considering other factors, such as cancer history, cancer treatment, or social determinants of health (SDOH), except for the PREVENT score, which includes the social deprivation index (SDI) in the risk model. While the PCE risk score does emphasize the role of risk enhancing factors and clinician–patient risk discussion, these traditional risk scores developed to predict ASCVD and CVD in the general population may provide inaccurate predictions for the cancer population; thus, hindering the appropriate allocation of resources to this vulnerable population.^10-14^

To address this gap, new cancer-specific risk scores must be developed that consider not only demographic characteristics, clinical risk factors, and laboratory results but also a patient’s cancer history, treatment history, and relevant neighborhood-level and individual-level SDOH. In this study, we aimed to develop cancer-specific scores that health-care providers can use to predict the 10-year CVD risk and compare it to conventional CVD scores in patients with breast cancer (BC), colorectal cancer (CRC), or lung cancer (LC).

## Methods

### Study setting

The study was conducted at the University Hospitals (UH) Seidman Cancer Center in Northeast Ohio, a hybrid academic-community tertiary care center serving urban, suburban, and rural populations.^15^^,^^16^ The center is part of an integrated network comprising 23 hospitals, over 50 health centers and outpatient facilities, and more than 200 physician offices across 16 counties in the region.^16^^,^^17^

### Data source

Data were collected from the UH repository, which is an open-source, web-based cancer data management system that consolidates various distinct data sources, providing high-quality and comprehensive longitudinal information for each patient.^15^^,^^16^^,^^18-20^ The available data includes both clinical and operational records from 2005 to the present, covering all aspects of care such as demographics, individual-level SDOH, comorbidities, outcomes, patient-reported outcomes, prescriptions, orders, pathology, lab values, pharmacy, radiology, radiation, genomics, hospitalizations, and encounters. All data in the UH repository undergo continuous review for completeness and accuracy.

Patient records were deidentified, and the study was approved by the UH of Cleveland Institutional Review Board (IRB).

### Study design and cohort

The study was designed as a retrospective cohort using secondary data. The cohort included patients aged 18 years or older diagnosed with BC, CRC, or LC between January 1, 2005 and March 31, 2012, providing a follow-up of 10 years until the data collection date (March 31, 2022). There was no minimum follow-up duration required for each individual. Patients with unknown diagnosis dates, unknown last follow-up dates, or unknown death dates were excluded. Male patients with BC were excluded.

### Outcomes

The primary outcome was 10-year CVD, defined as a composite of the diagnosis of new-onset non-fatal HF, non-fatal ischemic stroke (IS), and non-fatal myocardial infarction (MI), determined using International Classification of Diseases (ICD)-9 and ICD-10 codes (Table S1).^7^^,^^21^ The cause of death data was not available in our dataset, thus fatal cases of CVD were not included in the models. Time-to-event was calculated by subtracting the cancer diagnosis date from the CVD diagnosis date, while follow-up duration was determined by subtracting the cancer diagnosis date from the date of the patient’s last contact or death, as recorded in the electronic health record (EHR).

### Covariates

Demographic information, comorbidities, laboratory or vital values, tumor characteristics, treatment details, and census tract and individual-level SDOH data were collected for all eligible patients (Table S2).

The social deprivation index (SDI) was obtained linking the patient zip code with SDI derived from the American Community Survey.^22^ Individual-level SDOH were obtained from LexisNexis, the world’s largest electronic database for legal and public records-related information. We included individual-level SDOH across 4 domains (Table S2): social and community context, neighborhood and built environment, education access and quality, and economic stability.^16^^,^^23^

### Descriptive analysis

For each cancer type analyzed in our study, we summarized population characteristics by presenting categorical covariates as absolute numbers and percentages. Continuous covariates were assessed using histograms and the Kolmogorov–Smirnov test to determine their distribution. Covariates with a normal distribution were summarized using the mean and SD, while those with a nonnormal distribution were summarized using the median and quartiles.

### Machine learning-based cancer-specific CVD score

**Figure 1.**
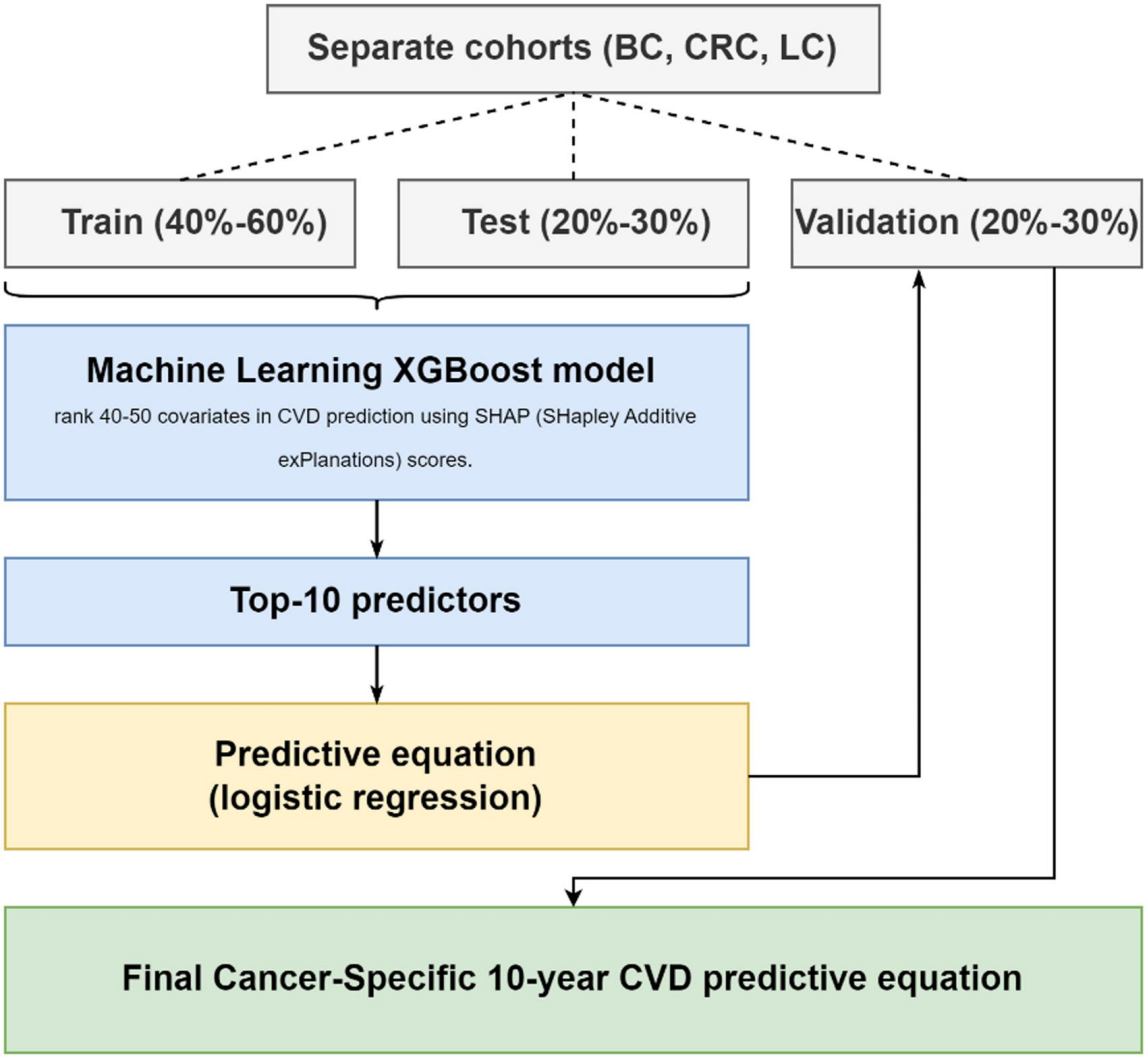
Study methodology for developing cancer-specific 10-year CVD predictive equations using a machine learning model to filter covariates. Abbreviations: BC = breast cancer; CRC = colorectal cancer; CVD = cardiovascular disease; LC = lung cancer.

The CVD scores developed in our study were composed of 2 phases: a machine learning (ML)-based algorithm for feature selection and a logistic regression to provide predictive equations (Figure 1).

The ML-based feature selection (described in Supplementary Methods) utilized the Extreme Gradient Boosting (XGBoost) algorithm, adapted for survival modeling outcomes (from the R package “mlr3proba”), with the aim of incorporating time-to-CVD in this phase to provide more accurate results.^16^^,^^24^

The top 10 CVD predictors for each cancer type, obtained from the ML algorithms, were then included in a multivariable logistic regression, with 10-year CVD as the outcome, in the validation subsets. The decision to limit the final regressions to the top 10 predictors aimed to develop generalized equations that maintain simplicity while remaining practical for health-care practitioners during patient encounters. This ensures the equations can be assessed and applied within a reasonable time frame, similar to existing CVD risk calculators, without omitting important predictors. The performance of the regressions was measured using the area under the curve (AUC) of the time-dependent receiver operating characteristic curve, ranging from 0 to 1.^25^^,^^26^ A 95% confidence interval (CI) was calculated through bootstrapping with 100 iterations. The intercept and coefficients from the logistic regression were used to create the CVD score equation, represented as *y = e*^(^^*b*^^0 + ^^*ba + …. + bz*^^)^/1 + *e^(b0 + ba + …. + bz^*^)^, where *y* is the 10-year CVD probability, *e* is the base of natural logarithms, *b0* is the intercept term, and *ba* to *bz* are the coefficients for each feature.^27^ For each CVD score, we reported the percentage of patients categorized within the following risk profiles: <5%, 5%-7.4%, 7.5%-10%, and >10%. These thresholds may vary depending on the specific score and are commonly used to categorize patients into low, borderline, intermediate, or high-risk groups, as well as to guide the initiation and intensity of prevention therapies.^28^

### Comparison to traditional CVD scores

The actual 10-year CVD risks for each cancer type were calculated using Kaplan–Meier and reported as mean risk with its 95% CI. Predicted 10-year CVD risk and AUC were then obtained from the AHA/ACC PCE, SCORE2, PREVENT simple, PREVENT simple + HbA1c, PREVENT simple + urine albumin-creatinine ratio (UACR), PREVENT simple + SDI, and PREVENT enhanced scores within the analyzed cohorts.^7^^,^^9^^,^^29^ PREVENT enhanced is the version of the PREVENT risk model which has all the variables included (PREVENT simple + HbA1c + UACR + SDI).

### Subgroup and sensitivity analysis

The performances of the cancer-specific and conventional scores were also analyzed for the ASCVD outcome (a composite of nonfatal myocardial infarction and nonfatal ischemic stroke), as the AHA/ACC PCE and SCORE2 were not originally designed to address CVD. For this comparison, the PREVENT-specific ASCVD equations were used. The performances were also compared for the HF outcome in the cancer-specific and PREVENT scores, as this is the outcome that distinguishes the cancer-specific and PREVENT equations from the AHA/ACC PCE and SCORE2. This comparison also provided insight into how much of the performance improvement was attributable to HF prediction.

A sensitivity analysis was conducted on patients without a cardiac history (defined as myocardial infarction, cardiomyopathy, stroke, or transient ischemic attack) at baseline to mitigate any potential bias introduced by this high-risk profile. A second sensitivity analysis was performed by excluding patients with advanced TNM stages of disease (III and IV), as this population has a shorter life expectancy and could also bias the model’s predictions.

## Results

We included 10 339 patients. Of those, 5687 (55.5%) had BC, 1605 (15.6%) had CRC, and 3047 (29.7%) had LC.

### Breast cancer

In the cohort of females with BC (Table 1), the median follow-up was 3346 days (interquartile range [IQR] = 1981-4370), and the median age was 60 years (IQR = 50-71). A positive smoking history was reported by 33% of the population, 19% had a diagnosis of diabetes, 54% had hypertension, and 36% had dyslipidemia. Advanced stage of cancer disease was diagnosed in 15% of the patients, 44% received chemotherapy, 29% received radiotherapy, 1% received immunotherapy, and 60% received endocrine therapy. TNM stage was the covariate with the highest rate of missingness (16.6%).

**Table 1.** Patient characteristics by cancer type: breast, colorectal, and lung cancer.

	Breast cancer	Colorectal cancer	Lung cancer
	*n* = 5687	*n* = 1605	*n* = 3047
**Population characteristics**			
Age—median (IQR), y	60 (50-71)	68 (57-77)	69 (60-77)
Female sex—*n* (%)	5687 (100)	864 (54)	1565 (51)
Race—*n* (%)			
Black	1018 (18)	274 (17)	487 (16)
White	4273 (75)	1130 (70)	1919 (63)
Non-Hispanic	396 (7)	201 (12)	641 (21)
Other	5132 (90)	1446 (90)	2628 (86)
Positive smoking history—*n* (%)	903 (33)	211 (13)	313 (10)
Diabetes—*n* (%)	1079 (19)	39 (2)	219 (7)
Hypertension—*n* (%)	3064 (54)	93 (5)	489 (16)
Dyslipidemia—*n* (%)	2070 (36)	45 (3)	504 (16)
Advanced cancer stage (III/IV)—*n* (%)	852 (15)	637 (40)	1806 (59)
Chemotherapy—*n* (%)	2513 (44)	767 (48)	1527 (50)
Radiotherapy—*n* (%)	1663 (29)	199 (12)	1146 (37)
Immunotherapy—*n* (%)	68 (1)	24 (1)	10 (0.3)
Endocrine therapy—*n* (%)	3413 (60)	—	—

Abbreviation: IQR = interquartile range.

The CVD rate in females with BC was 29.7% (20.7% HF; 8.8% MI; 10.4% IS). The top 10 CVD predictors from the BC ML model (C-index = 0.81 [95% CI = 0.80 to 0.82]; Table S3) were (Table 2): hypertension (SHapley Additive exPlanations [SHAP] Score [SS] = 0.49), dyslipidemia (SS = 0.38), age at diagnosis (SS = 0.29), body mass index (BMI) (SS = 0.17), chronic kidney disease (CKD) (SS = 0.12), Black race (SS = 0.11), positive smoking history (SS = 0.08), mastectomy (SS = 0.08), number of household members (SS = 0.07), and annual income (SS = 0.07).

**Table 2.** Machine learning XGBoost model performance, measured via C-index, and the top 10 covariates ranked according to their SHAP (SHapley Additive exPlanations) values.

	Breast cancer		Colorectal cancer		Lung cancer	
	*n* = 5687		*n* = 1605		*n* = 3047	
Ranking	Covariate	SHAP	Covariate	SHAP	Covariate	SHAP
1	Hypertension	0.49	BMI	0.85	eGFR	1.18
2	Dyslipidemia	0.38	HDL-C	0.70	Dyslipidemia	0.43
3	Age at diagnosis	0.29	Age at diagnosis	0.32	BMI	0.35
4	BMI	0.17	Social deprivation index	0.28	Age at diagnosis	0.26
5	Chronic kidney disease	0.12	Median neighborhood house values	0.08	CKD	0.25
6	Black race	0.11	Chemotherapy	0.06	Advanced cancer stage	0.25
7	Positive smoking history	0.08	Annual income	0.06	Previous cardiomyopathy	0.24
8	Undergone mastectomy	0.08	Total cholesterol	0.06	HbA1c	0.21
9	Number of household members	0.07	Neighborhood median income	0.05	Total cholesterol	0.20
10	Annual income	0.07	Number of properties owned	0.03	White race	0.15
C-index	0.81 (95% CI = 0.80 to 0.82)		0.71 (95% CI = 0.67 to 0.75)		0.86 (95% CI = 0.85 to 0.87)	

Abbreviations: BMI = body mass index; CKD = chronic kidney disease; eGFR = estimated glomerular filtration rate; HbA1c = hemoglobin A1c; HDL-C = high density lipoprotein cholesterol.

**Figure 2.**
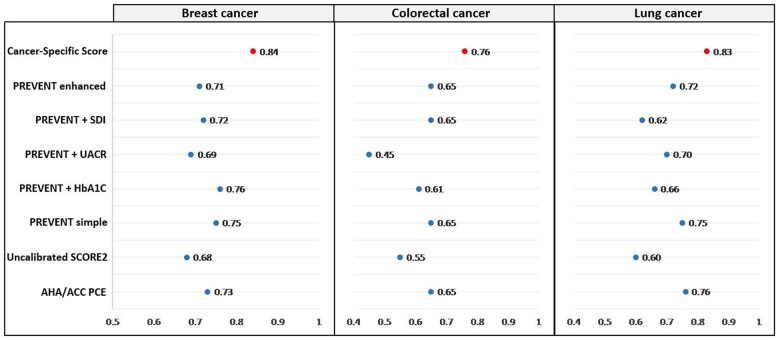
Representation of CVD prediction performance, measured by the AUC, for a cancer-specific score compared with conventional CVD scores in patients with breast cancer, colorectal cancer, or lung cancer. The horizontal axis corresponds to the AUC. Abbreviations: ACC = American College of Cardiology; AHA = American Heart Association; AUC = area under the curve; CI = confidence interval; CVD = cardiovascular disease; HbA1c = hemoglobin A1c; ML = machine learning; PCE = pooled cohort equation; PREVENT = Predicting Risk of cardiovascular disease EVENTs; SDI = social deprivation index; UACR = urine albumin creatinine ratio.

For CVD prediction, the AUC of the BC-specific score was 0.84 (Table 3; Tables S4 and S5). The AUCs of conventional CVD scores (Table 3; Figure 2) were 0.73 for AHA/ACC PCE, 0.68 for SCORE2, 0.75 for PREVENT simple, 0.76 for PREVENT + HbA1c, 0.69 for PREVENT + UACR, 0.72 for PREVENT + SDI, and 0.71 for PREVENT enhanced. Compared with the PREVENT + HbA1c, which had the second-best performance, the BC-specific score increased the percentage of patients with more than 10% CVD risk (Table S6).

**Table 3.** Comparison of machine learning-based CVD predictive model performance (AUC) vs traditional CVD scores for the CVD outcome.

	Breast cancer	Colorectal cancer	Lung cancer
	*n* = 5687	n = 1605	*n* = 3047
**ML-based equation time-dependent AUC**			
	0.84	0.76	0.83
**Conventional CVD scores AUC**			
AHA/ACC PCE	0.73	0.65	0.76
Uncalibrated SCORE2	0.68	0.55	0.60
PREVENT simple	0.75	0.65	0.75
PREVENT + HbA1c	0.76	0.61	0.66
PREVENT + UACR	0.69	0.45	0.70
PREVENT + SDI	0.72	0.65	0.62
PREVENT enhanced	0.71	0.65	0.72
**Real vs predicted 10-year CVD risk**			
Real CVD risk	21.3 (95% CI = 20.0 to 23.0)	9.8 (95% CI = 8.0 to 12.0)	27.6 (95% CI = 24.2 to 30.7)
AHA/ACC PCE	10.2 (95% CI = 9.6 to 10.9)	18.9 (95% CI = 16.6 to 21.1)	28.4 (95% CI = 25.3 to 31.6)
Uncalibrated SCORE2	11.1 (95% CI = 9.7 to 12.6)	22 (95% CI = 17.2 to 26.8)	19.8 (95% CI = 14.8 to 24.7)
PREVENT simple	14.9 (95% CI = 14.3 to 15.5)	19 (95% CI = 17.5 to 20.5)	29.3 (95% CI = 27.7 to 30.9)
PREVENT + HbA1c	17.4 (95% CI = 16.8 to 18.1)	16.5 (95% CI = 15.8 to 17.6)	26 (95% CI = 25.5 to 26.6)
PREVENT + UACR	19.3 (95% CI = 18.6 to 19.9)	25.6 (95% CI = 24.3 to 26.8)	37.8 (95% CI = 37.1 to 38.6)
PREVENT + SDI	28.5 (95% CI = 27.7 to 29.3)	34.7 (95% CI = 33.3 to 36.2)	53 (95% CI = 52.1 to 53.9)
PREVENT enhanced	13.3 (95% CI = 12.8 to 13.7)	18 (95% CI = 10.9 to 25.0)	40.7 (95% CI = 39.8 to 41.6)

Abbreviations: ACC = American College of Cardiology; AHA = American Heart Association; AUC = area under the curve; CI = confidence interval; CVD = cardiovascular disease; HbA1c = hemoglobin A1c; ML = machine learning; PCE = pooled cohort equation; PREVENT = Predicting Risk of cardiovascular disease EVENTs; SDI = social deprivation index; UACR = urine albumin creatinine ratio.

The BC-specific score also demonstrated the best performance among all scores after excluding patients with a cardiac history at baseline and those with advanced cancer (Tables S7 and S8). For ASCVD prediction (Table 4), the enhanced PREVENT score showed the best performance (AUC = 0.86). For HF prediction (Table 5), the BC-specific score and the PREVENT simple score both achieved the best performance (AUC = 0.76).

**Table 4.** Comparison of machine learning-based CVD predictive model performance (AUC) vs traditional scores for ASCVD as the outcome.

	Breast cancer	Colorectal cancer	Lung cancer
	*n* = 5687	*n* = 1605	*n* = 3047
**ML-based equation time-dependent AUC**			
	0.67	0.72	0.76
**Conventional ASCVD scores AUC**			
AHA/ACC PCE	0.74	0.58	0.70
Uncalibrated SCORE2	0.70	0.56	0.61
PREVENT simple	0.76	0.65	0.76
PREVENT + HbA1c	0.70	0.79	0.64
PREVENT + UACR	0.71	0.52	0.73
PREVENT + SDI	0.72	0.61	0.83
PREVENT enhanced	0.86	0.66	0.68
**Real vs predicted 10-year ASCVD risk**			
Real ASCVD risk	12.2 (95% CI = 11.1 to 13.3)	5.4 (95% CI = 3.8 to 7.0)	16.2 (95% CI = 13.4 to 18.9)
AHA/ACC PCE	10.2 (95% CI = 9.6 to 10.9)	16.8 (95% CI = 15 to 18.6)	18.8 (95% CI = 16.9 to 20.7)
Uncalibrated SCORE2	11.1 (95% CI = 9.7 to 12.6)	22 (95% CI = 17.2 to 26.8)	19.8 (95% CI = 14.8 to 24.7)
PREVENT simple	7.4 (95% CI = 7.1 to 7.7)	10.7 (95% CI = 10 to 11.4)	19.8 (95% CI = 19.5 to 20.1)
PREVENT + HbA1c	5.6 (95% CI = 5.4 to 5.9)	6.7 (95% CI = 6.3 to 7.0)	9.4 (95% CI = 9.3 to 9.6)
PREVENT + UACR	7.1 (95% CI = 6.8 to 7.4)	15 (95% CI = 14.2 to 15.8)	20.6 (95% CI = 20.1 to 21.1)
PREVENT + SDI	9.5 (95% CI = 9.2 to 9.8)	11.7 (95% CI = 11.2 to 12.2)	17.8 (95% CI = 17.4 to 18.1)
PREVENT enhanced	12.2 (95% CI = 10.3 to 14.2)	10 (95% CI = 9.5 to 10.5)	18.4 (95% CI = 13.8 to 23.1)

Abbreviations: ACC = American College of Cardiology; AHA = American Heart Association; AUC = area under the curve; CI = confidence interval; CVD = cardiovascular disease; HbA1c = hemoglobin A1c; ML = machine learning; PCE = pooled cohort equation; PREVENT = Predicting Risk of cardiovascular disease EVENTs; SDI = social deprivation index; UACR = urine albumin creatinine ratio.

**Table 5.** Comparison of machine learning-based CVD predictive model performance (AUC) vs traditional scores for heart failure as the outcome.

	Breast cancer	Colorectal cancer	Lung cancer
	*n* = 5687	*n* = 1605	*n* = 3047
**ML-based equation time-dependent AUC**			
	0.76	0.83	0.85
**Conventional scores 10-year HF risk AUC (95% CI)**			
PREVENT simple	0.76	0.72	0.76
PREVENT + HbA1c	0.70	0.59	0.64
PREVENT + UACR	0.68	0.58	0.71
PREVENT + SDI	0.70	0.71	0.67
PREVENT enhanced	0.70	0.55	0.76
**Real vs predicted 10-year HF risk**			
Real HF risk	15.2 (95% CI = 14 to 16.3)	6.8 (95% CI = 5.0 to 8.5)	22.5 (95% CI = 19.3 to 25.5)
PREVENT simple	12.2 (95% CI = 11.6 to 12.9)	12.6 (95% CI = 11.2 to 13.9)	25.6 (95% CI = 23.6 to 27.7)
PREVENT + HbA1c	21.6 (95% CI = 20.8 to 22.3)	26.3 (95% CI = 25.1 to 27.6)	40.8 (95% CI = 40.0 to 41.6)
PREVENT + UACR	23.4 (95% CI = 22.7 to 24.2)	38.1 (95% CI = 36.4 to 39.8)	56.1 (95% CI = 55.1 to 57.2)
PREVENT + SDI	17.8 (95% CI = 16.8 to 18.8)	15.4 (95% CI = 13.4 to 17.3)	15.1 (95% CI = 12.8 to 17.5)
PREVENT enhanced	16.3 (95% CI = 15.8 to 16.8)	27.9 (95% CI = 19.7 to 36.0)	28.6 (95% CI = 16.9 to 40.3)

Abbreviations: AUC = area under the curve; CI = confidence interval; CVD = cardiovascular disease; HbA1c = hemoglobin A1c; ML = machine learning; PCE = pooled cohort equation; PREVENT = Predicting Risk of cardiovascular disease EVENTs; SDI = social deprivation index; UACR = urine albumin creatinine ratio.

### Colorectal cancer

In the cohort of patients with CRC (Table 1), the median follow-up was 2110 days (IQR = 693-5318), and the median age was 68 years (IQR = 57-77). A positive smoking history was reported by 13% of the population, 2% had a diagnosis of diabetes, 5% had hypertension, and 3% had dyslipidemia. Advanced stage of cancer disease was diagnosed in 40% of the patients, 48% received chemotherapy, 12% received radiotherapy, and 1% received immunotherapy. TNM stage was the covariate with the highest rate of missingness (28.6%).

The CVD rate in patients with CRC was 4.9% (3.3% HF; 1.6% MI; 1.4% IS). The top 10 CVD predictors from the CRC ML model (C-index = 0.71 [95% CI 0.67-0.75]; Table S3) were (Table 2): BMI (SS = 0.85), high-density lipoprotein cholesterol (HDL-C; SS = 0.70), age at diagnosis (SS = 0.32), SDI (SS = 0.28), median neighborhood house values (SS = 0.08), chemotherapy (SS = 0.06), annual income (SS = 0.06), total cholesterol (SS = 0.06), neighborhood median income (SS = 0.05), and number of properties owned (SS = 0.03).

For CVD prediction, the AUC of the CRC-specific score was 0.76 (Table 3; Tables S4 and S5). The AUCs of conventional CVD scores (Table 3; Figure 2) were 0.65 for AHA/ACC PCE, 0.55 for SCORE2, 0.65 for PREVENT simple, 0.61 for PREVENT + HbA1c, 0.45 for PREVENT + UACR, 0.65 for PREVENT + SDI, and 0.65 for PREVENT enhanced. Compared with other scores, the CRC-specific score increased the percentage of patients with more than 10% CVD risk (Table S6).

The CRC-specific score also demonstrated the best performance among all scores after excluding patients with a cardiac history at baseline and those with advanced cancer TNM stages (Tables S7 and S8). For ASCVD prediction (Table 4), the PREVENT + HbA1c score showed the best performance (AUC = 0.79). For HF prediction (Table 5), the CRC-specific score achieved the best performance (AUC = 0.83).

### Lung cancer

In the cohort of patients with LC (Table 1), the median follow-up was 406 days (IQR = 153-1243), and the median age was 69 years (IQR = 60-77). A positive smoking history was reported by 10% of the population, 7% had a diagnosis of diabetes, 16% had hypertension, and 16% had dyslipidemia. Advanced stage of cancer disease was diagnosed in 59% of the patients, 50% received chemotherapy, 37% received radiotherapy, and 0.3% received immunotherapy. TNM stage was the covariate with the highest rate of missingness (15.8%).

The CVD rate in patients with LC was 9.2% (7.5% HF; 3.4% MI; 1.6% IS). The top 10 CVD predictors from to the LC ML model (C-index = 0.86 [95% CI 0.85-0.87]; Table S3) were (Table 2): estimated glomerular filtration rate (eGFR; SS = 1.18), dyslipidemia (SS = 0.43), BMI (SS = 0.35), age at diagnosis (SS = 0.26), CKD (SS = 0.25), advanced stage of cancer (SS = 0.25), previous cardiomyopathy (SS = 0.24), HbA1c (SS = 0.21), total cholesterol (SS = 0.20), and White race (SS = 0.15).

For CVD prediction, the AUC of the LC-specific score was 0.83 (Table 3; Tables S4 and S5). The AUCs of conventional CVD scores (Table 3; Figure 2) were 0.76 for AHA/ACC PCE, 0.60 for SCORE2, 0.75 for PREVENT simple, 0.66 for PREVENT + HbA1c, 0.70 for PREVENT + UACR, 0.62 for PREVENT + SDI, and 0.72 for PREVENT enhanced. Compared with PREVENT simple, which had the second-best performance, the LC-specific score increased the percentage of patients with more than 10% CVD risk (Table S6).

The LC-specific score also demonstrated the best performance among all scores after excluding patients with a cardiac history at baseline and those with advanced cancer TNM stages (Tables S7 and S8). For ASCVD prediction (Table 4), the PREVENT + SDI score showed the best performance (AUC = 0.83). For HF prediction (Table 5), the LC-specific score achieved the best performance (AUC = 0.85).

## Discussion

This study developed cancer-specific CVD risk scores to calculate 10-year CVD risk, including HF, by incorporating cancer-specific factors along with census tract and individual-level SDOH, in addition to commonly used covariates in traditional scores for the general population. Our results show that these cancer-specific risk scores outperform conventional scores in predicting 10-year CVD risk across the analyzed cancer types, with an increase of up to 0.31 points in the AUC. Our results suggest that conventional risk scores may inadequately capture CVD risk profiles of the cancer population.

Previous studies have tried to develop improved cardiovascular risk prediction models for cancer patients. Hippisley-Cox et al. developed sex-specific CVD risk score (the QRESEARCH cardiovascular risk algorithm [QRISK] 4) after identifying new risk factors including the history of brain cancer, blood cancer, lung cancer, or oral cancer.^30^ Strongman et al. also assessed whether including the cancer diagnosis would improve the QRISK3 cardiovascular risk score and concluded that cancer history should be considered in future risk models.^31^ Chow et al. developed a new HF risk score for adult survivors of pediatric cancers that included sex, age at diagnosis, chemotherapy, and radiotherapy.^32^ However, all these models always lacked at least 1 complete category of risk factors or were developed on the basis of previously developed models for the general population. For instance, the QRISK4 was based on the QRISK3 model,^33^ which was designed for the general population, and it only included limited number of cancers, excluding the most common cancers in men and women, and was not cancer-specific so ignoring unique pathophysiology of each cancer. The same observation was noted in the Strongman and Chow models.^31^^,^^32^ In addition, all these models lacked the inclusion of SDOH factors. This was further highlighted by a recent paper by McCracken et al. examining the predictive performance of 7 established risk scores in cancer survivors using date from the UK Biobank.^34^

With cancer-specific and SDOH factors included, our cancer-specific scores demonstrated superior performance to traditional scores when predicting CVD. The BC-specific CVD risk score, for example, achieved an AUC of 0.84, significantly higher than the best traditional risk scores PREVENT simple and PREVENT + HbA1c with AUCs of 0.75 and 0.76, respectively. Similar performance pattern was also found for CRC and LC-specific scores. The ML approach identified cancer-specific covariates, such as chemotherapy, surgical treatment, stage, and SDOH factors related to economic status, neighborhood conditions, and social support, as important predictors of CVD in cancer patients. In concordance with our findings, the literature demonstrates that various social and environmental factors play an important role in the development of CVD in cancer patients.^16^^,^^35^^,^^36^

The variation in the CVD predictors for each cancer type included in our study highlights the differences in the cancer biology for each cancer and the different factors, including cancer treatment received and social factors, that every cohort goes through in their cancer journey even before the initial diagnosis.^19^^,^^37-41^ For instance, in the LC cohort the top CVD predictors were all biologic rather than social factors and were mostly cardiometabolic factors plus kidney disease. On the other hand, in the BC cohort the top CVD predictors included previous cancer therapies received such as undergoing mastectomy. Also, BC and CRC, but not LC, cohorts included at least 2 SDOH factors in the top CVD predictors.

The increased performance of the cancer-specific scores for CVD prediction persisted even after excluding patients with advanced cancer stages or cardiac history, demonstrating the robustness and consistency of model. However, when predicting ASCVD, at least 1 traditional score outperformed the cancer-specific scores. This may be because our scores were primarily designed for CVD prediction. The superior performance of the cancer-specific scores in predicting HF supports this explanation. Additionally, when categorizing patients by their CVD risk scores, the BC and LC-specific scores identified a similar percentage of patients with more than  10% risk compared with the PREVENT models, which are the most current and highest performing CVD scores for the general population. The CRC score deviated from this pattern, likely due to the lower rate of CVD events in the CRC cohort, which is consistent with the lower performance of its score. Moreover, the CRC cohort exhibited a highest rate of missing data and included an older population with a lower prevalence of hypertension compared with the other cancer cohorts in our study, despite a similar or higher prevalence of this comorbidity being expected. This may reflect variations in missingness or data quality relative to the other cancer cohorts. These factors could have influenced model performance and should be considered when interpreting the results of our study.

From a clinical practice perspective, our work provides usable risk scores that health-care practitioners can apply to stratify CVD risk in patients with BC, CRC, or LC. The included covariates can be quickly captured during patient interviews. This work also highlights that current CVD risk scores used for the general population may not adequately capture risk in cancer patients. However, the developed scores and presented results must undergo independent and external validation before adoption into clinical practice. This validation can be performed retrospectively or prospectively by applying the equations to diverse, multicenter datasets, capturing the predictors included in the equations, and comparing their performance with both the results reported in our study and the existing CVD risk scores used in the general population. Until this validation is completed, the key message from this work is that incorporating cancer-specific covariates could improve CVD predictive performance for patients with cancer.

This study has several limitations. First, the use of data from a single health-care system may limit the generalizability of our results. In this design, it is difficult to estimate the extent to which performance is attributable to overfitting in this specific dataset or clinical environment. Second, there is inherent bias in using EHRs and ICD coding. For example, it is not possible to ensure that the appearance of a new ICD diagnosis code represents a new diagnosis rather than a preexisting condition that was not previously registered, nor can it be guaranteed that the registered date reflects the actual diagnosis date. In this case, the emergence of comorbidities may have been triggered by CVD rather than the other way around. Third, while we included a great number of variables that might be related to the development of CVD, other residual confounders might not be measured and could influence the CVD risk in cancer survivors. Fourth, due to data limitations, peripheral artery disease was not considered in the definitions of both ASCVD and CVD. Also, the cause of death was not available, thus fatal CVD occurring as first presentation of CVD was not accounted for in our risk models and competing risk modeling was not feasible. Fifth, our models did not account for current medications, including antidiabetic, antilipid, and antihypertensive therapies, due to data limitations. Lastly, it is important to note that the possibility for overfitting cannot be excluded as the derivation and validation were conducted on the same cohort.

Future studies should aim to externally validate these scores in more diverse cohorts of patients with different demographic, socioeconomic, and geographical settings to ensure robustness and generalizability. Also, including other tools such as coronary artery calcium (CAC) scoring in future models can significantly improve risk prediction.^42-44^ This becomes more relevant in cancer patients as they are more likely to get chest computed tomography scans, thus more likely to show incidental CAC that can serve for higher statin prescription to those in need.^45^

## Conclusion

Our study emphasizes the need for cancer-specific CVD risk prediction models and show superior performance of cancer-specific scores to the traditional risk scores in patients with BC, CRC, or LC. Our results underscore the importance of integrating cancer-related and SDOH covariates for precise CVD risk prediction and further guide primary prevention efforts and resources allocation in cancer survivors.

## Supplementary Material

pkaf016_Supplementary_Data

## Data Availability

University Hospitals (UH) Seidman Cancer Center database is available at University Hospitals Cleveland Medical Center and has access restricted to researchers with IRB approval.
