# Supplementary material for: A novel machine learning-based cancer-specific cardiovascular disease risk score among patients with breast, colorectal, or lung cancer
Source: JNCI Cancer Spectr. 2025 Jan 30;9(1):pkaf016. doi: 10.1093/jncics/pkaf016 (PMC11878632; doi:10.1093/jncics/pkaf016)
Supplement: pkaf016_Supplementary_Data [file pkaf016_supplementary_data.docx]

# Title: A novel machine learning-based cancer-specific CVD risk score among patients with breast, colorectal, or lung cancer.

# Supplementary Material

**Supplementary Methods.**

The data for each cancer cohort was partitioned chronologically as follows: 60% training, 20% testing, and 20% validation for BC and LC; 40% training, 30% testing, and 30% validation for CRC.^46^ These variations in data partitioning were adjusted to match the size of each cohort, ensuring adequate sizes for the training, testing, and validation cohorts necessary for subsequent cross-validation steps.^46^ The chronological partitioning was applied to prevent data leakage and reduce overfitting, bias, and limitations in the models.

The ML models were initially developed and trained on the training subset, with 10-year CVD as the outcome and time-to-CVD as the time-to-event. The models were then enhanced through hyperparameter tuning via the randomized search approach on the testing subset, employing 10-fold cross-validation with 100 iterations, prioritizing the concordance index (C-index), which was considered the performance metric and was reported together with its 95% confidence interval ^47,48^ The hyperparameters and their values used in each of the cancer-specific ML models are provided in **Supplementary Table 3**.^16^

From the final enhanced ML model for each cancer type, covariate importance scores for the predictors were obtained using SHapley Additive Explanations (SHAP) scores.^49,50^ These scores indicate how the model prediction changes when a particular feature is included, with higher SHAP scores meaning greater feature importance. In ML models, a prediction *f(x)* is represented as a constant base value plus the sum of the SHAP values: *f(x)=base value+sum of SHAP values*.^49,50^ Covariates were ranked according to their SHAP scores.^49,50^

**Supplementary Table 1**. International Classification of Diseases (ICD)-9 and -10 Codes Used to Define Outcomes and Covariates.

| **Outcome** | **ICD-9 and ICD-10 codes** |
| --- | --- |
| **10-year CVD*** | |
| **Heart Failure** | 428.xx, 398.9, 402.xx, 401.xx, 414.8, 425.xx, 785.51, I50.xx, I09.9, I11.0, I13.0, I13.2, I25.2, I42.0, I42.5–I42.9, I43.xx, R570 |
| **Ischemic Stroke** | 431.xx, 435.0x, 435.1x, 435.2x, 435.3x, 435.8x, 435.9x, 433.01, 433.11, 433.21, 433.31, 433.81, 433.91, 434.01, 434.11, 434.91, 997.01, 344.60, 344.61, I619, G45x, I63, I66, G834, G9781 |
| **Myocardial Infarction** | 411.xx, 410.xx, I20.0, I21.x, I24.x |
| **Comorbidities** | |
| **Chronic Kidney Disease** | 585.xx, N18x |
| **Dyslipidemia** | 272.x, E78x |
| **Diabetes** | 272, E78x |
| **Hypertension** | 401.1, 401.9, I10x |

CVD: cardiovascular diseases

* The selected ICD-9 and ICD-10 codes were based on previous literature and refined with the primary goal of capturing new-onset non-fatal cases for each of the listed composite CVD outcomes.

**Supplementary Table 2.** Covariates available for the analysis and their nature.

| Covariate name | Covariate nature | Categories (if categorical) | Cohorts with the covariate |
| --- | --- | --- | --- |
| Sex | Categorical | Male; Female | Breast Cancer; Colorectal Cancer; Lung Cancer |
| Age at diagnosis | Continuous | - | Breast Cancer; Colorectal Cancer; Lung Cancer |
| Self-reported Race | Categorical | Black; White; Other | Breast Cancer; Colorectal Cancer; Lung Cancer |
| Self-reported Ethnicity | Categorical | Hispanic; non-Hispanic | Breast Cancer; Colorectal Cancer; Lung Cancer |
| Date of Death | Date | - | Breast Cancer; Colorectal Cancer; Lung Cancer |
| Self-reported Smoking Status | Categorical | Current; Former; Never Smoker | Breast Cancer; Colorectal Cancer; Lung Cancer |
| Cancer Diagnosis Date | Date | - | Breast Cancer; Colorectal Cancer; Lung Cancer |
| Histology | Categorical | DCIS; LCIS; Ductal; Other | Breast Cancer; Colorectal Cancer; Lung Cancer |
| Vital status | Categorical | Live; Death | Breast Cancer; Colorectal Cancer; Lung Cancer |
| Surgical treatment for cancer date | Date | - | Breast Cancer; Colorectal Cancer; Lung Cancer |
| Surgical treatment for cancer | Categorical | Yes; No | Breast Cancer; Colorectal Cancer; Lung Cancer |
| Mastectomy Date | Date | - | Breast Cancer |
| Mastectomy | Categorical | Yes; No | Breast Cancer |
| Lumpectomy Date | Date | - | Breast Cancer |
| Lumpectomy | Categorical | Yes; No | Breast Cancer |
| Radiotherapy Start Date | Date | - | Breast Cancer; Colorectal Cancer; Lung Cancer |
| Radiotherapy | Categorical | Yes; No | Breast Cancer; Colorectal Cancer; Lung Cancer |
| Radiotherapy Right Breast | Categorical | Yes; No | Breast Cancer |
| Radiotherapy Left Breast | Categorical | Yes; No | Breast Cancer |
| Chemotherapy start date | Date | - | Breast Cancer; Colorectal Cancer; Lung Cancer |
| Chemotherapy | Categorical | Yes; No | Breast Cancer; Colorectal Cancer; Lung Cancer |
| Immunotherapy start date | Date | - | Breast Cancer; Colorectal Cancer; Lung Cancer |
| Immunotherapy | Categorical | Yes; No | Breast Cancer; Colorectal Cancer; Lung Cancer |
| Endocrine therapy start date | Date | - | Breast Cancer |
| Endocrine therapy | Categorical | Yes; No | Breast Cancer |
| TNM Stage | Categorical | 0, I, II, III, IV | Breast Cancer; Colorectal Cancer; Lung Cancer |
| Charlson Score | Continuous | - | Breast Cancer; Colorectal Cancer; Lung Cancer |
| Elixhauser Score | Continuous | - | Breast Cancer; Colorectal Cancer; Lung Cancer |
| Last Follow Up Date | Date | - | Breast Cancer; Colorectal Cancer; Lung Cancer |
| HER2 agents | Categorical | Yes; No | Breast Cancer |
| Aromatase Inhibitors | Categorical | Yes; No | Breast Cancer |
| LHRH Agonists | Categorical | Yes; No | Breast Cancer |
| Anthracyclines | Categorical | Yes; No | Breast Cancer |
| Non-anthracycline chemotherapy | Categorical | Yes; No | Breast Cancer |
| ER Antagonists | Categorical | Yes; No | Breast Cancer |
| PIK3CA mTOR inhibitors | Categorical | Yes; No | Breast Cancer |
| Newer Therapies | Categorical | Yes; No | Breast Cancer |
| HER2 | Categorical | Positive; Negative | Breast Cancer |
| ER | Categorical | Positive; Negative | Breast Cancer |
| PR | Categorical | Positive; Negative | Breast Cancer |
| Date of Death (if death) | Date | - | Breast Cancer; Colorectal Cancer; Lung Cancer |
| Heart Failure Diagnosis Date | Date | - | Breast Cancer; Colorectal Cancer; Lung Cancer |
| Heart Failure | Categorical | Yes; No | Breast Cancer; Colorectal Cancer; Lung Cancer |
| Ischemic Stroke Diagnosis Date | Date | - | Breast Cancer; Colorectal Cancer; Lung Cancer |
| Ischemic Stroke | Categorical | Yes; No | Breast Cancer; Colorectal Cancer; Lung Cancer |
| Myocardial Infarction Diagnosis Date | Date | - | Breast Cancer; Colorectal Cancer; Lung Cancer |
| Previous Cardiomyopathy | Categorical | Yes; No | Breast Cancer; Colorectal Cancer; Lung Cancer |
| Previous Coronary Artery Disease | Categorical | Yes; No | Breast Cancer; Colorectal Cancer; Lung Cancer |
| Previous Myocardial Infarction | Categorical | Yes; No | Breast Cancer; Colorectal Cancer; Lung Cancer |
| Previous Carotid Disease | Categorical | Yes; No | Breast Cancer; Colorectal Cancer; Lung Cancer |
| Previous Transient Ischemic Attack | Categorical | Yes; No | Breast Cancer; Colorectal Cancer; Lung Cancer |
| Hypertension | Categorical | Yes; No | Breast Cancer; Colorectal Cancer; Lung Cancer |
| Chronic Kidney Disease | Categorical | Yes; No | Breast Cancer; Colorectal Cancer; Lung Cancer |
| Dyslipidemia | Categorical | Yes; No | Breast Cancer; Colorectal Cancer; Lung Cancer |
| Obesity | Categorical | Yes; No | Breast Cancer; Colorectal Cancer; Lung Cancer |
| Diabetes | Categorical | Yes; No | Breast Cancer; Colorectal Cancer; Lung Cancer |
| Marital Status | Categorical | Married; Not married | Breast Cancer; Colorectal Cancer; Lung Cancer |
| Address Stability Index | Continuous | - | Breast Cancer; Colorectal Cancer; Lung Cancer |
| Own current property | Categorical | Yes; No | Breast Cancer; Colorectal Cancer; Lung Cancer |
| Neighborhood burglary index | Continuous | - | Breast Cancer; Colorectal Cancer; Lung Cancer |
| Neighborhood car theft index | Continuous | - | Breast Cancer; Colorectal Cancer; Lung Cancer |
| Neighborhood crime index | Continuous | - | Breast Cancer; Colorectal Cancer; Lung Cancer |
| Neighborhood median income | Continuous | - | Breast Cancer; Colorectal Cancer; Lung Cancer |
| Neighborhood murder index | Continuous | - | Breast Cancer; Colorectal Cancer; Lung Cancer |
| Neighborhood median home values | Continuous | - | Breast Cancer; Colorectal Cancer; Lung Cancer |
| Attended college | Categorical | Yes; No | Breast Cancer; Colorectal Cancer; Lung Cancer |
| Education Institution Rating | Continuous | - | Breast Cancer; Colorectal Cancer; Lung Cancer |
| Annual Income | Continuous | - | Breast Cancer; Colorectal Cancer; Lung Cancer |
| Owns a property | Categorical | Yes; No | Breast Cancer; Colorectal Cancer; Lung Cancer |
| Wealth index | Continuous | - | Breast Cancer; Colorectal Cancer; Lung Cancer |
| Number of properties Owned | Continuous | - | Breast Cancer; Colorectal Cancer; Lung Cancer |
| Household Income | Continuous | - | Breast Cancer; Colorectal Cancer; Lung Cancer |
| Number of household members | Continuous | - | Breast Cancer; Colorectal Cancer; Lung Cancer |
| Number of elderly household members | Continuous | - | Breast Cancer; Colorectal Cancer; Lung Cancer |
| Number of middle-aged household members | Continuous | - | Breast Cancer; Colorectal Cancer; Lung Cancer |
| Number of teenager household members | Continuous | - | Breast Cancer; Colorectal Cancer; Lung Cancer |
| Number of young adults household members | Continuous | - | Breast Cancer; Colorectal Cancer; Lung Cancer |
| Closest relatives distance in miles | Continuous | - | Breast Cancer; Colorectal Cancer; Lung Cancer |
| Number of transportation properties owned | Continuous | - | Breast Cancer; Colorectal Cancer; Lung Cancer |
| % appointments attended | Continuous | - | Breast Cancer; Colorectal Cancer; Lung Cancer |
| Total Cholesterol | Continuous | - | Breast Cancer; Colorectal Cancer; Lung Cancer |
| HDL | Continuous | - | Breast Cancer; Colorectal Cancer; Lung Cancer |
| SBP | Continuous | - | Breast Cancer; Colorectal Cancer; Lung Cancer |
| DBP | Continuous | - | Breast Cancer; Colorectal Cancer; Lung Cancer |
| Weight | Continuous | - | Breast Cancer; Colorectal Cancer; Lung Cancer |
| Height | Continuous | - | Breast Cancer; Colorectal Cancer; Lung Cancer |
| BMI | Continuous | - | Breast Cancer; Colorectal Cancer; Lung Cancer |
| eGFR | Continuous | - | Breast Cancer; Colorectal Cancer; Lung Cancer |
| HBa1C | Continuous | - | Breast Cancer; Colorectal Cancer; Lung Cancer |
| UACR | Continuous | - | Breast Cancer; Colorectal Cancer; Lung Cancer |
| Social Deprivation Index | Continuous | - | Breast Cancer; Colorectal Cancer; Lung Cancer |

**Supplementary Table 3.** XGBoost Machine Learning Model Hyperparameters for Each Cancer-Specific Model.

| Hyperparameter | Breast Cancer | Colorectal Cancer | Lung Cancer |
| --- | --- | --- | --- |
| nrounds | 4050 | 1050 | 3550 |
| nthread | 16 | 4 | 2 |
| verbose | 0 | 0 | 0 |
| eta | 0.1105636 | 0.1518549 | 0.2340255 |
| max_depth | 1 | 8 | 6 |
| min_child_weight | 0.609341 | 5.858873 | 0.0784552 |
| gamma | 3.985754 | 3.988856 | 4.566244 |
| subsample | 0.9760623 | 0.9258253 | 0.784552 |
| colsample_bytree | 0.7619377 | 0.9544351 | 0.9367749 |

The following hyperparameters were tuned: nrounds (the number of additional trees or weak learners added to the model), nthread (the number of parallel threads used), eta (the shrinkage of feature weights in each boosting step), max_depth (the maximum depth of each tree), min_child_weight (the minimum weight or number of samples required to create a new node in the tree), gamma (the minimum loss reduction required to create a new tree split), subsample (the fraction of observations or rows to subsample at each step), and colsample_bytree (the percentage of features or columns used to build each tree). These hyperparameters were tuned via the randomized search approach on the testing subset, employing 10-fold cross-validation with 100 iterations, aiming to maximize the concordance index (C-index), which was considered the performance metric

**Supplementary Table 4.** Features and its coefficients of the Cancer-Specific ML logistic regression equation for 10-Year CVD Prediction.

| Features | Breast Cancer | Colorectal Cancer | Lung Cancer |
| --- | --- | --- | --- |
| 10-year CVD risk = exp[b+w1​*x1​+w2​*x2​+…+w10​*x10​] / 1+exp[b+w1​*x1​+w2​*x2​+…+w10​*x10​]​ | | | |
| Intercept (b) | -5.425 | -1.964 | -7.127 |
| Hypertension (1=yes/0=no) | 1.156 | NA | NA |
| Dyslipidemia (1=yes/0=no) | 1.018 | NA | 0.827 |
| Age at diagnosis | 0.028 | 0.248 | 0.03 |
| BMI | 0.003 | 0.086 | 0.003 |
| CKD (1=yes/0=no) | 1.319 | NA | 1.599 |
| Black race (1=yes/0=no) | 0.441 | NA | NA |
| Positive smoking history (1=yes/0=no) | 0.545 | NA | NA |
| Mastectomy (1=yes/0=no) | 0.51 | NA | NA |
| Number of household members | 0.065 | NA | NA |
| Annual income (thousand dollars) | -0.006 | -0.0001 | NA |
| HDL-C | NA | 0.078 | NA |
| SDI | NA | -0.008 | NA |
| Median neighborhood home values (dollars) | NA | 0.00003 | NA |
| Chemotherapy (1=yes/0=no) | NA | 0.029 | NA |
| Total cholesterol | NA | -0.015 | 0.003 |
| Median neighborhood income (dollars) | NA | -0.00006 | NA |
| Number of properties owned | NA | -0.86 | NA |
| eGFR | NA | NA | 0.04 |
| Advanced stage of cancer (1=yes/0=no) | NA | NA | 0.014 |
| Cardiomyopathy history (1=yes/0=no) | NA | NA | 5.302 |
| HbA1C | NA | NA | 0.094 |
| White race (1=yes/0=no) | NA | NA | -0.077 |

BMI: body mass index; CKD: chronic kidney disease; CVD: cardiovascular disease; HDL-C: high density lipoprotein cholesterol; eGFR: estimated Glomerular Filtration Rate; SDI: social deprivation Index; HbA1C: hemoglobin A1C.

**Supplementary Table 5.** 95% Confidence Intervals for Model Performance (AUC), Calculated Using 100 Bootstrap Iterations.

|  | **Breast Cancer** | | | **Colorectal Cancer** | | | **Lung Cancer** | | |
| --- | --- | --- | --- | --- | --- | --- | --- | --- | --- |
|  | **CVD** | **ASCVD** | **HF** | **CVD** | **ASCVD** | **HF** | **CVD** | **ASCVD** | **HF** |
| **AHA/ACC PCE** | 0.69-0.76 | 0.68-0.77 | - | 0.57-0.74 | 0.43-0.75 | - | 0.65-0.79 | 0.59-0.76 | - |
| **Uncalibrated SCORE2** | 0.65-0.71 | 0.63-0.71 | - | 0.47-0.64 | 0.47-0.64 | - | 0.52-0.63 | 0.53-0.69 | - |
| **PREVENT simple** | 0.72-0.78 | 0.71-0.78 | 0.72-0.77 | 0.52-0.72 | 0.46-0.67 | 0.68-0.80 | 0.66-0.80 | 0.67-0.83 | 0.69-0.81 |
| **PREVENT + HbA1C** | 0.75-0.78 | 0.67-0.73 | 0.67-0.72 | 0.51-0.78 | 0.77-0.82 | 0.47-0.75 | 0.58-0.74 | 0.62-0.79 | 0.58-0.75 |
| **PREVENT + UACR** | 0.68-0.72 | 0.68-0.73 | 0.66-0.71 | 0.39-0.60 | 0.43-0.67 | 0.47-0.75 | 0.63-0.85 | 0.70-0.76 | 0.68-0.77 |
| **PREVENT + SDI** | 0.70-0.75 | 0.69-0.74 | 0.68-0.73 | 0.57-0.71 | 0.41-0.72 | 0.61-0.77 | 0.42-0.72 | 0.80-0.86 | 0.66-0.78 |
| **PREVENT enhanced** | 0.59-0.83 | 0.76-0.94 | 0.60-0.77 | 0.57-0.72 | 0.56-0.81 | 0.47-0.57 | 0.68-0.76 | 0.57-0.75 | 0.64-0.81 |
| **Cancer-specific equation** | 0.81-0.87 | 0.55-0.70 | 0.70-0.85 | 0.60-0.82 | 0.60-0.80 | 0.73-0.88 | 0.82-0.84 | 0.70-0.82 | 0.82-0.87 |
| **Removing patients with previous history of CVD** | | | | | | | | | |
| **AHA/ACC PCE** | 0.69-0.76 | - | - | 0.53-0.72 | - | - | 0.64-0.85 | - | - |
| **Uncalibrated SCORE2** | 0.63-0.72 | - | - | 0.48-0.61 | - | - | 0.50-0.70 | - | - |
| **PREVENT simple** | 0.72-0.78 | - | - | 0.60-0.74 | - | - | 0.69-0.90 | - | - |
| **PREVENT + HbA1C** | 0.65-0.77 | - | - | 0.55-0.83 | - | - | 0.69-0.81 | - | - |
| **PREVENT + UACR** | 0.67-0.72 | - | - | 0.37-0.65 | - | - | 0.63-0.83 | - | - |
| **PREVENT + SDI** | 0.69-0.74 | - | - | 0.57-0.81 | - | - | 0.61-0.81 | - | - |
| **PREVENT enhanced** | 0.62-0.77 | - | - | 0.60-0.71 | - | - | 0.64-0.79 | - | - |
| **Cancer-specific equation** | 0.76-0.84 | - | - | 0.70-0.79 | - | - | 0.80-0.90 | - | - |
| **Removing patients with advanced stage cancer** | | | | | | | | | |
| **AHA/ACC PCE** | 0.67-0.77 | - | - | 0.43-0.79 | - | - | 0.70-0.82 | - | - |
| **Uncalibrated SCORE2** | 0.64-0.74 | - | - | 0.50-0.65 | - | - | 0.57-0.72 | - | - |
| **PREVENT simple** | 0.70-0.80 | - | - | 0.60-0.71 | - | - | 0.71-0.83 | - | - |
| **PREVENT + HbA1C** | 0.72-0.78 | - | - | 0.54-0.80 | - | - | 0.55-0.75 | - | - |
| **PREVENT + UACR** | 0.66-0.73 | - | - | 0.45-0.66 | - | - | 0.69-0.79 | - | - |
| **PREVENT + SDI** | 0.69-0.75 | - | - | 0.58-0.73 | - | - | 0.44-0.69 | - | - |
| **PREVENT enhanced** | 0.55-0.73 | - | - | 0.57-0.82 | - | - | 0.73-0.93 | - | - |
| **Cancer-specific equation** | 0.78-0.88 | - | - | 0.67-0.79 | - | - | 0.81-0.91 | - | - |

ACC: American college of cardiology; AHA: American heart association; AUC: area under the curve; CI: confidence intervals; CVD: cardiovascular disease; HbA1c: Hemoglobin A1c; ML: machine learning; PCE: pooled cohort equation; PREVENT: Predicting Risk of cardiovascular disease EVENTs; SDI: social deprivation index; UACR: urine albumin creatinine ratio.

**Supplementary Table 6.** The percentage of patients within risk categories for each model predicting CVD for each cancer type.

|  | Breast Cancer | | | | Colorectal Cancer | | | | Lung Cancer | | | |
| --- | --- | --- | --- | --- | --- | --- | --- | --- | --- | --- | --- | --- |
|  | **Risk Threshold** | | | | | | | | | | | |
|  | **<5%** | **5-7.4%** | **7.5-10%** | **>10%** | **<5%** | **5-7.4%** | **7.5-10%** | **>10%** | **<5%** | **5-7.4%** | **7.5-10%** | **>10%** |
| Cancer-Specific  ML-based Score | 39.9 | 10.1 | 6.9 | 43.1 | 0 | 0 | 0 | 100 | 7.1 | 0 | 7.1 | 85.7 |
| AHA/ACC PCE | 43.3 | 9.6 | 8.2 | 38.9 | 16.6 | 8.9 | 7.6 | 66.9 | 7.8 | 5.0 | 3.5 | 83.7 |
| Uncalibrated SCORE2 | 78.9 | 2.5 | 1.7 | 16.9 | 63.5 | 4.3 | 3.4 | 28.8 | 66.8 | 3.6 | 2.1 | 27.5 |
| PREVENT simple | 23.1 | 9.5 | 9.8 | 57.5 | 9.4 | 8.6 | 9.4 | 72.5 | 3.1 | 8.3 | 4.7 | 83.9 |
| PREVENT + HbA1C | 56.8 | 1.8 | 1.9 | 39.5 | 47.3 | 3.6 | 2.8 | 46.4 | 21.8 | 2.1 | 1.7 | 74.4 |
| PREVENT + UACR | 51.9 | 2.3 | 1.8 | 43.9 | 35.5 | 2.5 | 2.9 | 59.1 | 13.6 | 1.4 | 1.3 | 83.7 |
| PREVENT + SDI | 46.4 | 1.9 | 1.7 | 50.0 | 30.0 | 1.1 | 1.4 | 67.6 | 11.7 | 5.0 | 5.0 | 87.4 |
| PREVENT enhanced | 52.0 | 3.3 | 3.0 | 41.7 | 19.8 | 6.4 | 4.4 | 69.5 | 11.9 | 2.2 | 1.7 | 84.2 |

ACC: American college of cardiology; AHA: American heart association; AUC: area under the curve; CI: confidence intervals; CVD: cardiovascular disease; HbA1c: Hemoglobin A1c; ML: machine learning; PCE: pooled cohort equation; PREVENT: Predicting Risk of cardiovascular disease EVENTs; SDI: social deprivation index; UACR: urine albumin creatinine ratio.

**Supplementary Table 7. S**ensitivity analysis showing the performance of the various models predicting CVD after removing patients with previous history of CVD (n= 8,948).

|  | **Breast Cancer** | **Colorectal Cancer** | **Lung Cancer** |
| --- | --- | --- | --- |
|  | **n=4,785** | **n=1,542** | **n=2,621** |
| **ML-based equation Time-Dependent AUC** | | | |
|  | 0.79 | 0.75 | 0.86 |
| **Conventional CVD scores AUC (95% CI)** | | | |
| **AHA/ACC PCE** | 0.73 | 0.65 | 0.78 |
| **Uncalibrated SCORE2** | 0.68 | 0.56 | 0.60 |
| **PREVENT simple** | 0.76 | 0.70 | 0.82 |
| **PREVENT + HbA1C** | 0.75 | 0.63 | 0.70 |
| **PREVENT + UACR** | 0.69 | 0.50 | 0.70 |
| **PREVENT + SDI** | 0.71 | 0.67 | 0.76 |
| **PREVENT enhanced** | 0.70 | 0.63 | 0.74 |
| **Real vs predicted 10-year CVD risk** | | | |
| **Real CVD risk** | 11 (9.8-12.1) | 6.8 (5.0-8.6) | 10.8 (8.1-13.5) |
| **AHA/ACC PCE** | 8 (7.4-8.7) | 16.1 (14.1-18.1) | 22.9 (17.9-27.8) |
| **Uncalibrated SCORE2** | 7.9 (6.4-9.4) | 21.1 (16.0-26.2) | 22.9 (13.8-32.1) |
| **PREVENT simple** | 11.9 (11.3-12.5) | 18.2 (16.6-19.8) | 17.7 (15.5-19.9) |
| **PREVENT + HbA1C** | 19.8 (19.0-20.5) | 17.3 (16.4-18.3) | 29 (28.5-29.5) |
| **PREVENT + UACR** | 20.7 (20.0-21.4) | 26.3 (25.1-27.6) | 40.6 (39.8-41.3) |
| **PREVENT + SDI** | 31.1 (30.2-32.0) | 35.8 (34.3-37.3) | 57.4 (56.6-58.2) |
| **PREVENT enhanced** | 14.2 (13.7-14.7) | 18.7 (10.7-26.8) | 43.1 (42.2-44.0) |

ACC: American college of cardiology; AHA: American heart association; AUC: area under the curve; CI: confidence intervals; CVD: cardiovascular disease; HbA1c: Hemoglobin A1c; ML: machine learning; PCE: pooled cohort equation; PREVENT: Predicting Risk of cardiovascular disease EVENTs; SDI: social deprivation index; UACR: urine albumin creatinine ratio.

**Supplementary Table 8.** Sensitivity analysis showing the performance of the various models predicting CVD after removing patients with advanced stage of cancer (n= 7,044).

|  | **Breast Cancer** | **Colorectal Cancer** | **Lung Cancer** |
| --- | --- | --- | --- |
|  | **n=4,835** | **n=968** | **n-1,241** |
| **ML-based equation Time-Dependent AUC** | | | |
|  | 0.80 | 0.75 | 0.84 |
| **Conventional CVD scores AUC** | | | |
| **AHA/ACC PCE** | 0.74 | 0.69 | 0.80 |
| **Uncalibrated SCORE2** | 0.71 | 0.59 | 0.63 |
| **PREVENT simple** | 0.78 | 0.68 | 0.77 |
| **PREVENT + HbA1C** | 0.76 | 0.59 | 0.68 |
| **PREVENT + UACR** | 0.69 | 0.49 | 0.73 |
| **PREVENT + SDI** | 0.71 | 0.63 | 0.61 |
| **PREVENT enhanced** | 0.71 | 0.71 | 0.82 |
| **Real vs predicted 10-year CVD risk** | | | |
| **Real CVD risk** | 21.3 (19.8-22.7) | 11.2 (8.5-13.8) | 29 (25.2-32.7) |
| **AHA/ACC PCE** | 10.5 (9.8-11.2) | 17.2 (14.8-19.6) | 30.7 (27.2-34.3) |
| **Uncalibrated SCORE2** | 11.3 (9.7-12.9) | 22.4 (16.8-28.1) | 19.5 (14.1-24.9) |
| **PREVENT simple** | 15.4 (14.8-16.1) | 20.3 (18.5-22.1) | 29.8 (27.9-31.7) |
| **PREVENT + HbA1C** | 17.3 (16.6-18) | 15.3 (14.1-16.5) | 19.8 (18.8-20.9) |
| **PREVENT + UACR** | 19.2 (18.5-19.9) | 24.1 (22.6-25.7) | 31.5 (30.1-32.8) |
| **PREVENT + SDI** | 28.2 (27.3-29.1) | 32.2 (30.4-34.0) | 42.1 (40.5-43.7) |
| **PREVENT enhanced** | 13.1 (12.6-13.6) | 18.2 (9.6-26.8) | 34 (32.5-35.5) |

ACC: American college of cardiology; AHA: American heart association; AUC: area under the curve; CI: confidence intervals; CVD: cardiovascular disease; HbA1c: Hemoglobin A1c; ML: machine learning; PCE: pooled cohort equation; PREVENT: Predicting Risk of cardiovascular disease EVENTs; SDI: social deprivation index; UACR: urine albumin creatinine ratio.
